# *Biosensors* Best Paper Award 2015

**DOI:** 10.3390/bios5020364

**Published:** 2015-06-23

**Authors:** Jeff D. Newman

**Affiliations:** Cranfield Biotechnology Centre, SATM, Building 39, Cranfield University, Beds MK43 0AL, UK; E-Mail: j.d.newman@cranfield.ac.uk

With the start of 2015, *Biosensors* is instituting an annual award to recognize outstanding papers related to science and technology of biosensors and biosensing that meet the aims, scope and high standards of this journal. We are pleased to announce the first “*Biosensors* Best Paper Award” for 2015. The winners were chosen by the Editor-in-Chief and a group of Editorial Board Members of *Biosensors* from among all the papers published in 2013 and 2014 to track citations. Only full research articles were considered. We gladly announce that the following 3 papers were awarded the *Biosensors* Best Paper Award in 2015:

1^st^ Prize
**Linda G. Lee, Eric S. Nordman, Martin D. Johnson and Mark F. Oldham**
A Low-Cost, High-Performance System for Fluorescence Lateral Flow Assays
*Biosensors* **2013**, *3*(4), 360-373; doi:10.3390/bios3040360
Available online: http://www.mdpi.com/2079-6374/3/4/360
2^nd^ Prize
**Scherrine Tria, Leslie H. Jimison, Adel Hama, Manuelle Bongo and Róisín M. Owens**
Sensing of EGTA Mediated Barrier Tissue Disruption with an Organic Transistor
*Biosensors* **2013**, *3*(1), 44-57; doi:10.3390/bios3010044
Available online: http://www.mdpi.com/2079-6374/3/1/44
3^rd^ Prize
**Petri Ihalainen, Himadri Majumdar, Tapani Viitala, Björn Törngren, Tuomas Närjeoja, Anni Määttänen, Jawad Sarfraz, Harri Härmä, Marjo Yliperttula, Ronald Österbacka and Jouko Peltonen**
Application of Paper-Supported Printed Gold Electrodes for Impedimetric Immunosensor Development
*Biosensors* **2013**, *3*(1), 1-17; doi:10.3390/bios3010001
Available online: http://www.mdpi.com/2079-6374/3/1/1

These three exceptional papers are valuable contributions to *Biosensors* and the biosensing field. On behalf of the Prize Awarding Committee and the Editorial Board of *Biosensors*, we would like to congratulate these three teams for their excellent work. In recognition of their accomplishment, Drs. Linda G. Lee, Róisín M. Owens and Petri Ihalainen will be awarded the privilege of publishing an additional research paper free of charge in open access format in *Biosensors*.

Prize Awarding Committee
**Prof. Dr. Luigi Campanella**
  Environment, Cultural Heritage and Analytical Chemistry
  University of Rome La Sapienza
  Vecchio Edificio Chimica, P.le Aldo Moro, 5 - 00185 - Roma, Italy
**Prof. Dr. Nicole Jaffrezic-Renault**
  Institute of Analytical Sciences, UMR CNRS 5280
  Department LSA, 5 Rue de La Doua, 69100 Villeurbanne, France
  Tel. +33472448306; Fax: +33 472 43 12 06
  Website: http://www.univ-lyon1.fr
**Prof. Dr. Ashok Mulchandani**
  Department of Chemical and Environmental Engineering
  University of California
  Riverside, CA 92521, USA
  Fax: +1-951-827-5696
  Website: http://www.engr.ucr.edu/~adani/
**Dr. Jeff D. Newman**
  Biomedical Engineering Centre
  School of Engineering
  Vincent Building, Cranfield University, Bedford, MK43 0AL, UK
  Website: http://www.cranfield.ac.uk/health/abouttheschool/people/page8129.html
**Prof. Dr. Michael Thompson**
  Department of Chemistry
  University of Toronto
  80 St. George Street, Toronto, Ontario, M5S 3H6, Canada
  Fax: +1 416 978 8775
  Website: http://www.chem.utoronto.ca/staff/THOMPSON/

